# An optimized support vector machine for lung cancer classification system

**DOI:** 10.3389/fonc.2024.1408199

**Published:** 2024-12-23

**Authors:** Mayowa O. Oyediran, Olufemi S. Ojo, Ibrahim A. Raji, Abidemi Emmanuel Adeniyi, Oluwasegun Julius Aroba

**Affiliations:** ^1^ Department of Computer Engineering, Ajayi Crowther University, Oyo, Nigeria; ^2^ Department of Computer Sciences, Ajayi Crowther University, Oyo, Nigeria; ^3^ Department of Mathematical and Computer Sciences Education, Emmanuel Alayande University of Education, Oyo, Nigeria; ^4^ Department of Computer Science, Bowen University, Iwo, Nigeria; ^5^ Honorary Research Associate, Department of Operations and Quality Management, Durban University of Technology, Durban, South Africa; ^6^ Centre for Ecological Intelligence, Faculty of Engineering and the Build Environment (FEBE), Electrical and Electronic Engineering Science, University of Johannesburg, Johannesburg, South Africa

**Keywords:** chameleon swarm algorithm (CSA), lung cancer, support vector machine, optimization techniques, machine learning

## Abstract

**Introduction:**

Lung cancer is one of the main causes of the rising death rate among the expanding population. For patients with lung cancer to have a higher chance of survival and fewer deaths, early categorization is essential. The goal of thisresearch is to enhance machine learning to increase the precision and quality of lung cancer classification.

**Methods:**

The dataset was obtained from an open-source database and was utilized for testing and training. The suggested system used a CT scan picture as its input image, and it underwent a variety of image processing operations, including segmentation, contrast enhancement, and feature extraction.

**Results:**

The training process produces a chameleon swarm-based supportvector machine that can identify between benign, malignant, and normal nodules.

**Conclusion:**

The performance of the system is evaluated in terms of false-positive rate (FPR), sensitivity, specificity, recognition time and recognition accuracy.

## 1 Introduction

Due to its higher death rate than the combined deaths from breast, colon, and prostate cancer, lung cancer is one of the deadliest diseases in the world (1). The primary cause of carcinoma or lung cancer is cigarette smoking. Inhaling second-hand smoke, using pipes or cigars, being exposed to radon or asbestos at work or home, and having a family history of the disease are additional risk factors for lung cancer (1).

Malignant nodules in the lung that have the potential to spread quickly throughout the body after entering the bloodstream are referred to as lung cancer. Due to its rapid spread after creation, lung cancer poses a greater threat to life than other tumors (2). According to a previous study (3), exhaustion, chest discomfort, coughing, hemoptysis, sore throat, shortness of breath, weariness, weight loss, and chest infection are common signs of lung cancer.

Preventing the growth and spread of cancer cells is largely dependent on the early diagnosis process. While CT scan imaging can reveal both suspected and unsuspected lung cancer nodules, it is not the most reliable imaging technique for lung cancer diagnosis (4).

The clever behavior of chameleons in the wild served as the inspiration for the development of the chameleon swarm algorithm (CSA) in recent years (5). Many researchers have expressed interest in using it in various fields due to its simplicity and ease of implementation (6). It has been applied in image segmentation (7) and power engineering (8, 9). It has also been proven to be successful in resolving optimization issues in biology (10) and mathematics (11), respectively.

One machine learning approach used for classification is support vector machine (SVM). Assigning points to one of two disjoint half spaces is a common method of classifying points (12, 13). To classify data, SVMs project low-dimensional vectors into a high-dimensional space and create ideal hyperplanes that make it simple to classify fresh data points in the future (14). The linearly indistinguishable problem is converted into a linearly divisible problem in the high-dimensional space by selecting an appropriate kernel function (15). Hence, this study developed a model to select appropriate parameters for SVM using a chameleon swarm optimization technique.

A computer-aided diagnosis (CADx) system for classifying lung nodules was evaluated by (16). The main areas of focus were (i) the utility of the traditional CADx system (produced imaging feature + machine learning algorithm); (ii) a comparison of machine learning algorithms, namely, gradient tree boosting (XGBoost) vs. SVM; and (iii) the efficacy of parameter optimization using Bayesian optimization and random search. To calculate the feature vector, a local binary pattern (LBP) variation was employed. A feature vector and its matching label were used to train an SVM or XGBoost. SVM and XGBoost parameters were optimized using Bayesian techniques using the Tree Parzen Estimator (TPE). For TPE comparison, a random search was conducted. To optimize and assess the performance of our CADx system, we employed leave-one-out cross-validation. The receiver operating characteristic analysis’ area under the curve (AUC) was used to assess performance. Compared to random search, Bayesian optimization of the SVM and XGBoost parameters was more effective. Two board-certified radiologists had AUC values of 0.898 and 0.822, according to an observer study. According to the findings, our CADx system’s diagnostic precision in lung nodule classification was on par with that of radiologists.

The contribution of this study represents a novel approach to optimizing SVM parameters by using CSA to improve lung cancer classification performance through the CS-SVM model. Compared to conventional SVM, recognition accuracy, sensitivity as well as specificity, and recognition time are greatened with the introduction of CS-SVM technique using CSA in this study. This demonstrates how well optimization works in practice.

Şekeroğlu and Emirzade (17) developed and implemented an SVM-based computer-aided diagnosis (CAD) system for the identification of lung cancer. Their goal in developing the CAD system was to improve diagnosis accuracy while cutting down on diagnosis time. The 271 recorded whole-lung CT scan images that make up the Lung Image Database Consortium (LIDC) subset of the public database were used in this investigation. To lessen impulse noise, a median filter was utilized during image preparation. The suggested CAD system used edge detection, morphological segmentation, and global thresholding in conjunction with the binarization procedure. Otsu’s global image thresholding technique was utilized to convert the grayscale images into binary. Following the binarization procedure, the boundaries of the CT lung image were determined using the gradient detection approach. Then, extraneous perimeter lines were eliminated using the morphological operation. For every CT scan, statistical features were retrieved from the histogram and the gray-level co-occurrence matrix (GLCM) in four different orientations. Owing to the extracted features’ high dimension, feature selection procedures were used to increase the classifier’s accuracy. The SVM found the best-fit kernel for the classification by using its three most preferred kernels: linear, quadratic, and radial basis functions. In conclusion, the CAD system outperformed other suggested methods, achieving 97% accuracy, 92% sensitivity, and 97.3% specificity.

Sharma et al. (18) introduced a novel CAD system that uses image processing techniques to identify lung cancer nodules and classifies them using Convolutional Neural Network (CNN). The investigator utilized a CT-scan image as the input image and applied various image processing techniques, including segmentation, morphological operations, histogram equalization, and feature extraction. To identify if a nodule is malignant or not, the classifier was trained. The CAD system’s overall performance was enhanced by this strategy, which yielded 98.08% accuracy, 96.6% specificity, and 90.39% sensitivity analysis.

To diagnose lung cancer more accurately, Singh and Gupta (19) developed a model in which they used neural networks and multilayer perceptron. The four criteria that they have employed are recall, accuracy, F1 score, and precision. A total of 6,910 benign and 8,840 malignant lung cancer photos were among the 15,750 clinical images that they used for testing and training. Finally, they developed an 88.55% accuracy multilayer perceptron, which proved to be a superior classifier.

Four classification techniques—SVM, Back Propagation Neural Network (BPNN), Probabilistic neural network (PNN), and k-means clustering—were compared by (20). High-boost filtering was utilized during the proposition stage. The Fuzzy C- Mean (FCM) clustering algorithm was then used for segmentation, and statistical techniques were employed for feature extraction. SVM, PNN, BPNN, and K-means clustering had accuracy ratings of 85%, 82%, 86%, and 81%, in that order. Local ternary co-occurrence patterns (LTCoP) and LBP feature extraction variations were carried out for 50 pictures from LIDC by Bruntha et al. (21). SVM was utilized for classification, whereas median filtering, intensity thresholding, and segmentation were employed in the preposition stage. It was 91.5% for LTCoP and 89.2% for LBP. In addition, Karthiga and Rekha (22) conducted a comparison of the Fuzzy Multicategory Support Vector Machines Classifier (FMSVM), multilayer perceptron (MLP), K-Nearest Neighbor (KNN), Support Vector Machines (SVM), and I-Naive Bayesian Classifier (I-NBC) classifications; the corresponding accuracies were 98%, 52%, 46%, 96%, and 98%, respectively.

According to (23), machine learning algorithms including SVM, random forest, and artificial neural network (ANN) have been used to train the retrieved features. Then, to determine which technique has the best accuracy, a variety of factors like accuracy, precision, and recall were assessed, and, with the highest accuracy of 96%, the ANN was established as the best.

A method for identifying the condition using a classification technique called Content-Based Image Retrieval (CBIR)-based Bag of Features (BOF) and grouping pattern categories using K-means clustering was proposed by Bhatt and Soni (24). A testing accuracy of 98.56% and a training accuracy of 99% were achieved with this strategy. Yunianto et al. and Aroba et al. (25–29) examined a total of 120 CT scan image data, utilizing Otsu thresholding for segmentation and a median filter for placement. After that, data were retrieved using the GLCM feature, which had angle and direction fluctuations. Naïve Bayes was utilized for the classification procedure, and 88.33% accuracy was achieved.

## 2 Methodology

### 2.1 Image acquisition

The dataset acquired from open source database (www.kaggle.com) contained a total of 1,097 images. These images were grouped into three classes: normal, benign, and malignant, and the datasets for each of the classes were 416, 120, and 561, respectively. The CT scans were originally collected in DICOM format, and the whole dataset was divided into training and testing dataset using the K-fold validation method where K = 10.

### 2.2 Image pre-processing

This is a step that suppresses noise or other small fluctuations in an image and is also used to improve the interpretability or perception of information in an image to enhance better input when used in other image analysis techniques. In this study, contrast limited adaptive histogram equalization (CLAHE) was utilized to produce an enhanced version of the original image.

### 2.3 Segmentation

In this study, fuzzy C-means was used to perform segmentation by determining the cancer nodules in the lung (30). This phase helped to identify the regions of interest in the lung nodule for a better classification process.

### 2.4 Feature extraction

LBP is a simple yet influential algorithm used in this study for feature extraction. The algorithm works by analyzing the texture of an image, which is defined by the distribution of intensity variations in small regions of the image called local neighborhoods. The LBP algorithm works as follows:

- Select a pixel in the image and define a local neighborhood around it, typically a square or circular region of fixed size.
- Compare the intensity value of the central pixel with the intensity values of its neighbors. If a neighbor has a higher intensity value than the central pixel, then assign it a value of 1. Otherwise, assign it a value of 0.
- Concatenate the binary values of the neighbors into a binary string, creating a unique pattern for the local neighborhood.
- Repeat this process for every pixel in the image, creating an LBP image where each pixel is replaced by its corresponding binary pattern.
- Calculate a histogram of the LBP patterns over the entire image. This histogram represents the texture of the image and can be used as a feature descriptor.

### 2.5 Proposed technique

This study used the strength chameleon swarm optimization algorithms to improve the performance of the SVM. The steps involved in achieving this optimization technique are as follows (**Algorithm 1**):

Algorithm 1 **Chameleon swarm support vector machine.**

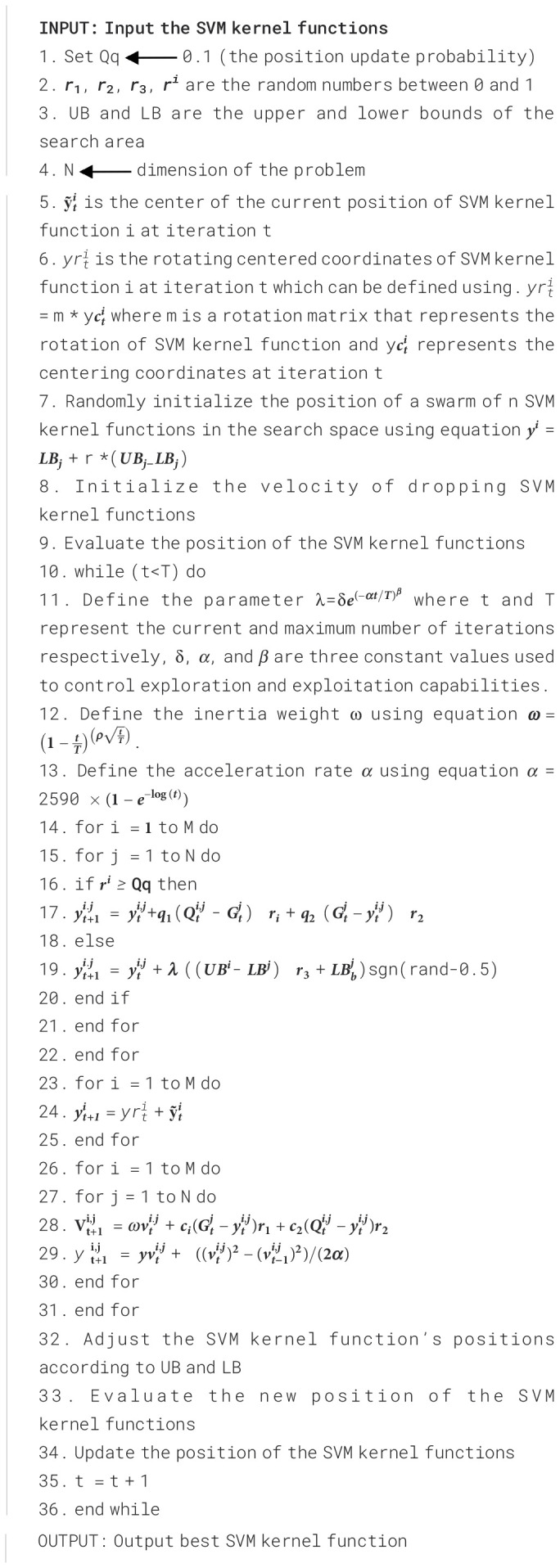

I. Chameleon swarm initialization: Initialize a population of chameleon individuals with random parameter settings (for example, *C* and kernel parameters for SVM).

II. Objective function evaluation: Evaluate the performance of each chameleon’s parameter settings using cross-validation and the SVM objective function.

III. Color-changing Behavior: Implement color-changing behavior to update chameleon parameters. For example, adjust the “C” value or kernel parameters based on the chameleon’s performance.

IV. Swarm movement: Allow chameleons to move within the parameter space, guided by the color-changing behavior and the optimization objectives.

V. Fitness evaluation: Re-evaluate the fitness of chameleons based on their updated parameter settings.

VI. Best chameleon selection: Select the best-performing chameleon as the optimal parameter setting for SVM.

### 2.6 Proposed design

This proposed design included all processes involved in a lung cancer classification system from image acquisition and finally classification. Acquired images went through pre-processing stage and feature extraction using LBP, and the formulated CS-SVM algorithm was incorporated to perform feature selection and classification. The design was implemented in the Matrix Laboratory (R2020a) system specification of 2.60-Ghz processor, 500-GB HDD (hard disk drive), 4 GB of RAM and 64-bit operating system on a Windows 10 platform. Graphical user interface was designed using tool box in a MATLAB development environment for simulating classification system.

## 3 Results and discussion

### 3.1 Analyzing results with the malignant dataset

The outcomes of the SVM and CS-SVM techniques applied to the performance measures utilizing malignant datasets are shown in **Table 1**. The SVM technique yielded a 5.95% false-positive rate (FPR), 96.69% sensitivity, 94.05% specificity, and 95.90% accuracy in 59.89 s. Similarly, in 39.87 s, the CS-SVM approach produced 97.33% accuracy, 97.71% sensitivity, 96.43% specificity, and a false-positive rate of 3.57%. According to the results in **Table 1**, the CS-SVM approach performed better than the SVM technique in terms of recognition accuracy, sensitivity, specificity, and false-positive rate.

**Table 1:** Findings using the SVM and CS-SVM methods on the malignant dataset.

Technique	FPR (%)	Specificity (%)	Sensitivity (%)	Recognition accuracy (%)	Recognition time (s)
**SVM**	5.95	94.05	96.69	95.90	59.89
**CS-SVM**	3.57	96.43	97.71	97.33	39.87

### 3.2 Analyzing results with the benign dataset

The findings for the SVM and CS-SVM approaches in terms of the performance metrics using Benign datasets are shown in **Table 2**. A false-positive rate of 11.11%, sensitivity of 91.67%, specificity of 88.89%, and accuracy of 90.83% at 59.92 s were all attained by the SVM technique. The CS-SVM method also produced results at 39.86 s with a false-positive rate of 5.56%, sensitivity of 94.05%, specificity of 94.44%, and recognition accuracy of 94.17%. According to the results in **Table 2**, the CS-SVM approach performed better than the SVM technique in terms of recognition accuracy, sensitivity, specificity, and false-positive rate.

**Table 2:** Results obtained by the SVM and CS-SVM techniques with the benign dataset.

Technique	FPR (%)	Specificity (%)	Sensitivity (%)	Recognition accuracy (%)	Recognition time (s)
**SVM**	11.11	88.89	91.67	90.83	59.92
**CS-SVM**	5.56	94.44	94.05	94.17	39.86

### 3.3 Analyzing results with the normal dataset

The outcomes of the SVM and CS-SVM techniques applied to the performance measures utilizing the normal dataset are shown in **Table 3**. A false-positive rate of 9.60%, sensitivity of 94.85%, specificity of 90.40%, and accuracy of 93.51% at 61.74 s were attained by the SVM technique. Similar results were obtained at 40.40 s with the CS-SVM technique: a false-positive rate of 6.40%, sensitivity of 96.22%, specificity of 93.60%, and recognition accuracy of 95.43%. According to the results in **Table 3**, the CS-SVM approach performed better than the SVM technique in terms of recognition accuracy, sensitivity, specificity, and false-positive rate.

**Table 3:** Results obtained by the SVM and CS-SVM techniques with the normal dataset.

Technique	FPR (%)	Specificity (%)	Sensitivity (%)	Recognition accuracy (%)	Recognition time (s)
**SVM**	9.60	90.40	94.85	93.51	61.74
**CS-SVM**	6.40	93.60	96.22	95.43	40.40

### 3.4 Comparative analysis of the results for all the datasets

The comparison analysis of the evaluation results obtained from both techniques shows that the developed CS-SVM outperformed SVM across all metrics. The false-positive rates for CS-SVM and SVM were 3.57% and 5.95% for malignant, 5.56% and 11.11% for benign, and 6.40% and 9.60% for normal, respectively. Also, the results of specificity for CS-SVM and SVM were 96.43% and 94.05% for malignant, 94.44% and 88.89% for benign, and 93.60% and 90.40% for normal, respectively. The CS-SVM and SVM produced sensitivity rates of 97.71% and 96.69% for malignant, 94.05% and 91.67% for benign, and 96.22% and 94.85% for normal, respectively. Furthermore, CS-SVM and SVM attained recognition accuracy of 97.33% and 95.90% for malignant, 94.17% and 90.83% for benign, and 95.43% and 93.51% for normal, respectively. In addition, recognition time for CS-SVM and SVM were 39.87 s and 59.89 s for malignant, 39.86 s and 59.92 s for benign, and 40.40 s and 61.74 s for normal, respectively. **Table 4** shows the summary of the comparison of both techniques with respect to the datasets.

**Table 4:** Combined results for CS-SVM and SVM with respect to the datasets.

	Malignant	Benign	Normal	Average
FPR (%)				
SVM	5.95	11.11	9.60	10.22
CS-SVM	3.57	5.56	6.40	5.18
Specificity (%)				
SVM	94.05	88.89	90.40	91.11
CS-SVM	96.43	94.44	93.60	94.82
Sensitivity (%)				
SVM	96.69	91.67	94.85	94.40
CS-SVM	97.71	94.05	96.22	95.99
Recognition accuracy (%)				
SVM	95.90	90.83	93.51	93.41
CS-SVM	97.33	94.17	95.43	95.64
Recognition time (s)				
SVM	59.89	59.92	61.74	60.52
CS-SVM	39.87	39.86	40.40	36.71

**Figure 1:**
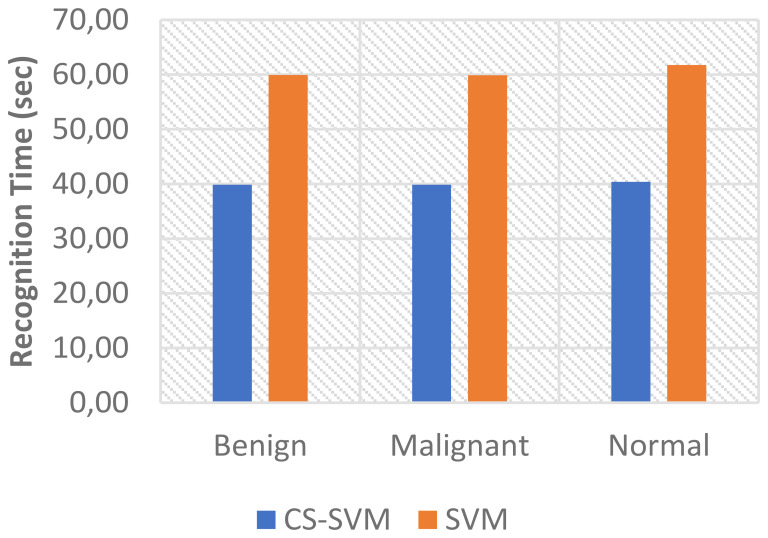
Comparison of recognition time for lung cancer classification system.

**Figure 2:**
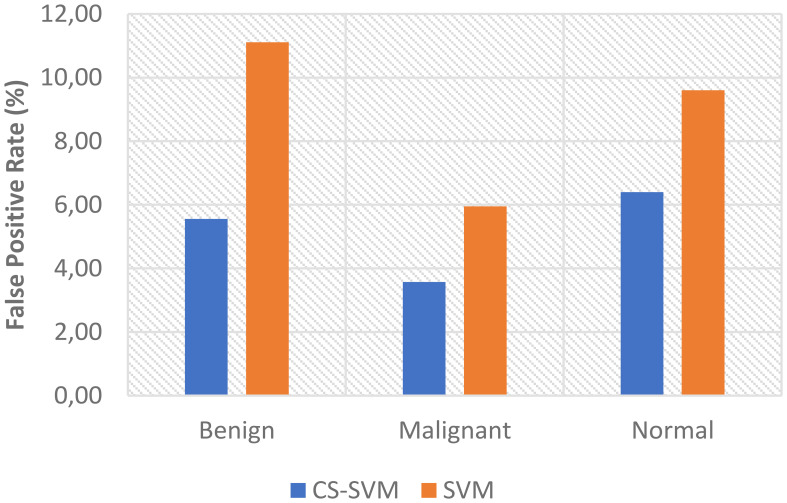
Comparison of FPR for lung cancer classification system.

**Figure 3:**
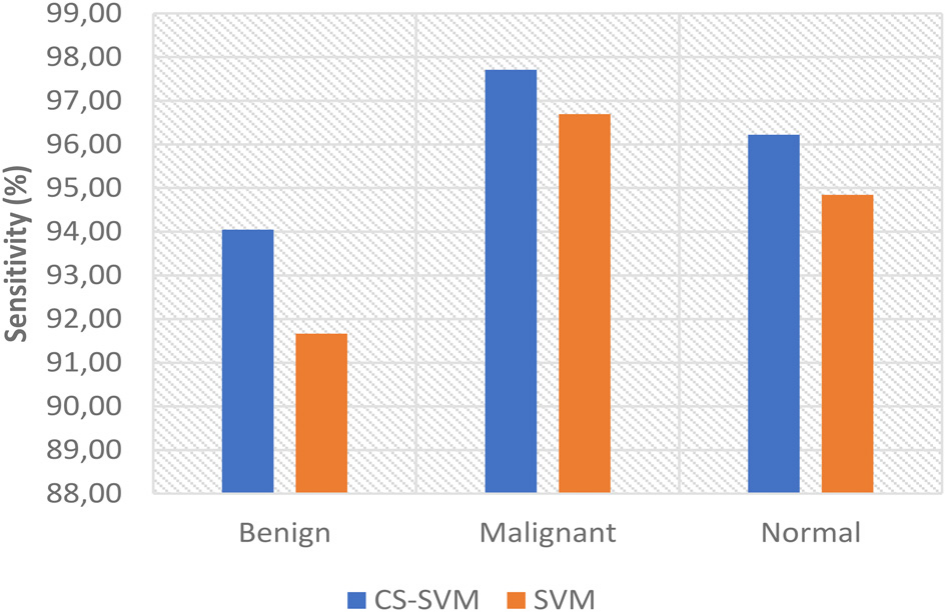
Comparison of sensitivity for lung cancer classification system.

**Figure 4:**
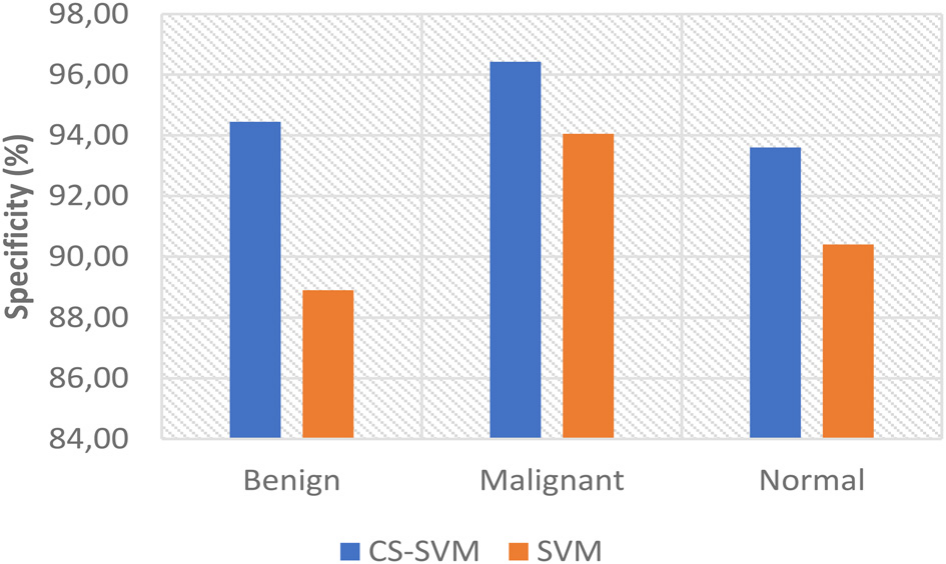
Comparison of specificity for lung cancer classification system.

**Figure 5:**
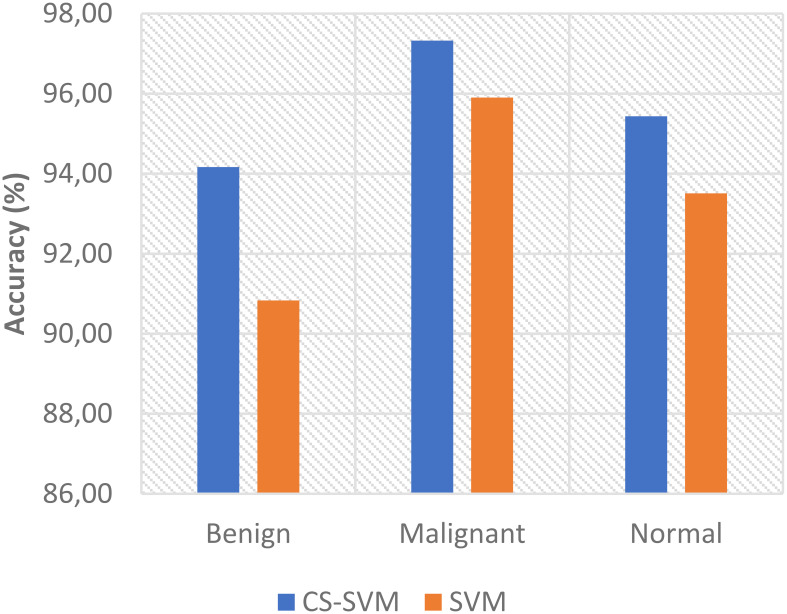
Comparison of recognition accuracy for lung cancer classification system.

The charts showing the comparison of each of the evaluation metrics for the lung cancer classification system are as follows (**Figures 1**–**5**):

### 3.5 Limitation of the study

This study uses a restricted dataset that does not entirely mirror the variability observed within clinical settings across different contexts. Differences in image quality, patient demographics, or clinical conditions could influence how broadly this study’s findings can be applied. Despite having many pictures, the particular dataset has fewer pictures relative to the numerous variations present in actual clinical situations.

### 3.6 Strength of the study

A robust methodology was used in the study, which employs advanced image processing techniques as well as the CSA used in optimizing parameters for SVMs. This strength is very important. The comparative analysis between SVMs and CS-SVMs is thorough when looking at different performance metrics. This is commendable and adds value to the study.

## 4 Conclusion

This research developed chameleon swarm–based SVM for lung cancer classification system. This conclusion explains why the developed technique performed better than the other method examined in this study in terms of recognition accuracy, false-positive rate, sensitivity, recognition time, and specificity. This serves as evidence that enhancing the performance of systems for the classification of lung cancer disease can be achieved more successfully with the use of the CS-SVM technique. By leveraging the optimal selection of SVM parameters through the CS-SVM approach, the method significantly reduced false positive rates while achieving higher classification accuracy.

### 4.1 Future scope

More feature extraction and fusion methods may be included in future studies for better results and system performance investigation. Furthermore, higher convergence optimization algorithms can be incorporated for comparison and understanding of which technique returns the best results in the context of cancer classification.

## Data Availability

The original contributions presented in the study are included in the article/supplementary material. Further inquiries can be directed to the corresponding authors.
